# Supplementary material for: Patterns of Intron Gain and Loss in Fungi
Source: PLoS Biol. 2004 Nov 30;2(12):e422. doi: 10.1371/journal.pbio.0020422 (PMC532390; doi:10.1371/journal.pbio.0020422)
Supplement: Table S1 — Also available at http://genes.mit.edu/NielsenEtAl/. (4.3 MB ZIP). [file pbio.0020422.st001.zip › NielsenEtAl/html/221.html]

AN3222.1.NCU01945.1.MG02955.1.FG11629.1


```
 CLUSTAL W (1.82) Multiple Sequence Alignments - Introns Inserted


Sequence 1: NCU01945.1	431 aa
Sequence 2: MG02955.1	436 aa
Sequence 3: FG11629.1	430 aa
Sequence 4: AN3222.1	444 aa
Alignment Length: 496 aa
Number Identitical Residues: 211 aa
Alignment Score (without introns) 10230


MG02955.1 	MVDYSFTSKIPPFENIDRLLAR~INQTE~WI~KG~LLAVPFVLYSQPTGVFEPSHGSG--
NCU01945.1	------MASLPPSCWMFLPARL~AESTE1WI~KA~LLAVPFVLYSQPHGVLEDPDRSVDT
FG11629.1 	----------------------~MTTRY~RV~EC1LLAVPFVLYSQPTGVFG--DGPS--
AN3222.1  	-------------MTTRYRVEF1SPKRP~AV1QG~LLAVPFVLHSQPTAVQEE-DATK--
          	                              : :  ********:*** .*    . .   

MG02955.1 	VSKMSEEAHRRYAEIMRDVETMIDDHSN2I~V~FHQNDENPFPSKLKLLVPSIGPFFTRL
NCU01945.1	LSQTREEAHRRYSEIFRDIEAMIDDH--~I1A~HQNDAENPFPSKLKLLVPSIGPFFTRL
FG11629.1 	VTQMAEEAHRRYAEIMRDVELMIDDH--~-~I1SRQQDDSMFPSKLRMLIPTAGPFFTRL
AN3222.1  	LAAVAHDTHQRYAEIFSDVERLLNDH--~-~I1DHELSGAAGKSKLKLLVPTVGTFFTRL
          	::   .::*:**:**: *:* :::**        ::       ***::*:*: *.*****

MG02955.1 	PLEAAFKFQDRKRYISSRRFVSPSFNDVRLILNSAQIMAVTTYGTLQLATFDGDVTLYDD
NCU01945.1	PLEAAFKFQDNKRYISSRRFVSPSFNDIRLILNSAQIMAVTTYGTLQLATFDGDVTLYDD
FG11629.1 	PLEAAFKYQDRKRYISSRRFVAPSFNDVRQILNSAQSMAVTN-GSLQLATFDGDVTLYDD
AN3222.1  	YLREAFDYQDRQRFISRRRFVAPSFNDVRLILNSAQLLGLVHTKGLELVTFDGDVTLYDD
          	 *. **.:**.:*:** ****:*****:* ****** :.:.    *:*.***********

MG02955.1 	GKSLEPDSPVIPRMMD1LLHKNVKIGIVTAAGYTTADRYYARLHGLLDSMASSSTLTDQQ
NCU01945.1	GQSLEPTSPVIPRLLD1LLRKNVKIGIVTAAGYTTADRYYSRLHGLLDAMANSADLTPSQ
FG11629.1 	GFNLEPTSPVIPRLLD1LLRKNIKIGIVTAAGYTSADRYYERLHGLLDAIAESTDLDPVQ
AN3222.1  	GANLNADSPVIPRIIR~LLQQGIKVGIVTAAGYTDEAKYYERLQGLLDVMRDASDLTDEQ
          	* .*:. ******::  **::.:*:*********   :** **:**** : .:: *   *

MG02955.1 	KQNLIVMGGEANYLFQFDSSAPHLLSAVPRERWLTPEMATWNEGDIKNMLDVAESALNDC
NCU01945.1	KQSLVVMGGEANYLFEFDSSSPHLLAPVPRQHWLTPEMAAWNEQDIAQLLDVAEAALRDC
FG11629.1 	KQSIIIMGGEANYLFEYSPSSPCKLAPVPRTQWLTPEMASWSDADITRLLDVAENALRDC
AN3222.1  	RSALIVMGGESNYLFRYDASSPHRLTYVPRENWVIGEMATWEEEDITQLLNIAESSLRAC
          	:. :::****:****.:..*:*  *: *** .*:  ***:*.: ** .:*::** :*. *

MG02955.1 	VKNLNLPATVMRKDRAVGIIPRDPDVRIARESLEETVLVVQKILELSTAG----------
NCU01945.1	IKTLNLPATLMRKDRAVGIIPVSPEIRIPRESLEETVLLVQKILELSTVG----------
FG11629.1 	VNNLNLPAMIMRKDRAVGIIPKTPGTRIARESLEETVLVVQRILELSSLGSSEERPTKHR
AN3222.1  	VANLNLPVSVLRKDRAVGVFPKNRG-RLSREQLEETVLVVQNTVERSQVG----------
          	: .****. ::*******::*     *:.**.******:**. :* *  *          

MG02955.1 	------------RAGRVPFCAFN-----------------------------------GG
NCU01945.1	------------RSRRVPFCAFN-----------------------------------GG
FG11629.1 	PSSPPIPPSVASQSRRVPFCAFN-----------------------------------GG
AN3222.1  	--------------SRLPFCAFNGCSPFFVSHYLRHHPPRRKYKPPIQPCMLISITQPGG
          	               *:******..:.   :      ..  . ... ...   : :..**

MG02955.1 	NDVFVDIGDKSWGVTVCQQWFAG-GSTDKFIRGENTLHVGDQFLSAGANDFR~ARSVGTT
NCU01945.1	RDVFVDIGDKSWGVTVCQRWFSQKEGPHGVIKGENTLHVGDQFLSAGANDFR~ARSVGTT
FG11629.1 	NDVFVDIGDKSWGVTVCQQWFGS-KENGGAIRGENTLHVGDQFLSAGSNDFK~ARSVGTT
AN3222.1  	NDVFVDIGDKSWGVRACQQYFGG-------IDPSRTLHVGDQFLSAGANDFK0ARLASTT
          	.************* .**::*.        *  ..************:***: ** ..**

MG02955.1 	AWIASPAETVELLDELAELMGKKLS
NCU01945.1	AWIASPVETVELLDELAELMGKKMS
FG11629.1 	AWIASPAETVELLDELADMMQKKLS
AN3222.1  	AWIASPAETVQLLDEL-DTIQKVLA
          	******.***:***** : : * ::
```
